# Comprehensive analysis of the prognosis, tumor microenvironment, and immunotherapy response of SDHs in colon adenocarcinoma

**DOI:** 10.3389/fimmu.2023.1093974

**Published:** 2023-03-06

**Authors:** Han Nan, Pengkun Guo, Jianing Fan, Wen Zeng, Chonghan Hu, Can Zheng, Bujian Pan, Yu Cao, Yiwen Ge, Xiangyang Xue, Wenshu Li, Kezhi Lin

**Affiliations:** ^1^School and Hospital of Stomatology, Wenzhou Medical University, Wenzhou, Zhejiang, China; ^2^School of Basic Medical Sciences, Wenzhou Medical University, Wenzhou, Zhejiang, China; ^3^School of Second Clinical Medical, Wenzhou Medical University, Wenzhou, Zhejiang, China; ^4^The First School of Medicine, School of Information and Engineering, Wenzhou Medical University, Wenzhou, China; ^5^Department of Hepatobiliary Surgery, Wenzhou Central Hospital, The Dingli Clinical Institute of Wenzhou Medical University, Wenzhou, China; ^6^Wenzhou Collaborative Innovation Center of Gastrointestinal Cancer in Basic Research and Precision Medicine, Wenzhou Key Laboratory of Cancer-related Pathogens and Immunity, Experiemtial Center of Basic Medicine, School of Basic Medical Sciences, Wenzhou Medical University, Wenzhou, China; ^7^Department of General Surgery, The Second Affiliated Hospital and Yuying Children’s Hospital of Wenzhou Medical University, Wenzhou, China; ^8^Institute of Molecular Virology and Immunology, School of Basic Medical Sciences, Wenzhou Medical University, Wenzhou, Zhejiang, China

**Keywords:** succinate dehydrogenase (SDH), colorectal cancer, colon adenocarcinoma (COAD), prognostic, immune infiltration, immune treatment

## Abstract

**Background:**

Succinate dehydrogenase (SDH), one of the key enzymes in the tricarboxylic acid cycle, is mainly found in the mitochondria. SDH consists of four subunits encoding SDHA, SDHB, SDHC, and SDHD. The biological function of SDH is significantly related to cancer progression. Colorectal cancer (CRC) is one of the most common malignant tumors globally, whose most common histological subtype is colon adenocarcinoma (COAD). However, the correlation between SDH factors and COAD remains unclear.

**Methods:**

The data on pan-cancer was obtained from The Cancer Genome Atlas (TCGA) database. Kaplan-Meier survival analysis showed the prognostic ability of SDHs. The cBioPortal database reflected genetic variations of SDHs. The correlation analysis was conducted between SDHs and mitochondrial energy metabolism genes (MMGs) and the protein-protein interaction (PPI) network was built. Consequently, Univariate and Multivariate Cox Regression Analysis on SDHs and other clinical characteristics were conducted. A nomogram was established. The ssGSEA analysis visualized the association between SDHs and immune infiltration. Immunophenoscore (IPS) explored the correlation between SDHs and immunotherapy, and the correlation between SDHs and targeted therapy was investigated through Genomics of Drug Sensitivity in Cancer. Finally, qPCR and immunohistochemistry detected SDHs’ expression.

**Results:**

After assessing SDHs differential expression in pan-cancer, we found that SDHB, SDHC, and SDHD benefit COAD patients. The cBioPortal database demonstrated that SDHA was the top gene in mutation frequency rank. Correlation analysis mirrored a strong link between SDHs and MMGs. We formulated a nomogram and found that SDHB, SDHC, SDHD, and clinical characteristics correlated with COAD patients’ survival. For T helper cells, Th2 cells, and Tem, SDHA, SDHB, SDHC, and SDHD were significantly enriched in the high expression group. Moreover, COAD patients with high SDHA expression were more suitable for immunotherapy. And COAD patients with different SDHs’ expression have different sensitivity to targeted drugs. Further verifying the gene and protein expression levels of SDHs, we found that the tissues were consistent with the bioinformatics analysis.

**Conclusions:**

Our study analyzed the expression and prognostic value of SDHs in COAD, explored the pathway mechanisms involved, and the immune cell correlations, indicating that SDHs might be biomarkers for COAD patients.

## Introduction

Colon adenocarcinoma (COAD) is the most common histological subtype, accounting for more than 90% of CRC (1). According to statistics, Colorectal cancer (CRC) ranks third among incident cases in both men and women and the third most lethal cancer worldwide in 2022 (2). New treatments for COAD have developed with advances in surgery and medicine, but long-term survival rates of patients remain considerably lower (3). Therefore, searching for potential cancer biomarkers and developing new-targeted drugs for immunotherapy may become essential research directions in COAD.

In recent years, tumor immunotherapy has been used in high-incidence malignancies such as colon cancer (4), non-small-cell lung (5), and triple-negative breast cancer (6), which would activate immunologic cells to attack the tumor by cell metabolic signaling pathway. In contrast to normal differentiated cells, which rely primarily on mitochondrial oxidative phosphorylation to generate the energy needed for cellular processes, most cancer cells instead rely on aerobic glycolysis, a phenomenon termed “the Warburg effect”. Mitochondria are the powerhouses of cells, and aerobic glycolysis is considered the primary metabolic phenotype of tumor cells, which meet the challenges of high energy demand for rapid cancer cell division and migration by enhancing glycolysis exhibited under aerobic conditions (7). In terms of tumor metabolism, enhanced glycolysis phenotype reflects the progression of tumor development (8). To illustrate, enhanced glycolysis regulates pancreatic cancer metastasis (9), and colorectal cancer metastasis (10). Additionally, a pan-cancer analysis of glycolysis with TCGA database regarded increased tumor glycolytic activity as inferior survival in various cancers (11).

A report indicated that succinate dehydrogenases (SDHs) are closely related to mitochondria and are primarily involved in the occurrence and progression of tumors (12, 13). Moreover, succinate, which accumulates as a result of SDH inhibition, inhibits HIF-α prolyl hydroxylases in the cytosol, stabilizes and activates Hypoxia-inducible factor-1 (HIF-1) (14). HIF-1, a transcription factor involves in hypoxic induction of glycolysis, leads to malignant transformation (15). SDH, also known as mitochondrial complex II, is composed of four subunits encoding SDHA, SDHB, SDHC, and SDHD (16). The structure of the protein comprises a hydrophilic head and a hydrophobic tail. The hydrophilic head protrudes into the mitochondrial matrix, and the hydrophobic tail anchors the protein to the mitochondrial inner membrane (17). SDH, functioning as the catalytic core, the head portion is composed of the flavoprotein SDHA and the iron sulphur (Fe-S) containing protein SDHB. The membrane domain comprises the SDHC and SDHD subunits, containing a bound heme moiety and a binding site for ubiquinone (17, 18).

There exists a close relationship between malignancies and the expression of succinate and SDH, including SDH mutations, regulation of mRNA expression, and cancer immunosurveillance (16). SDH mutations have been found in familial paragangliomas and pheochromocytomas (19–24), renal carcinomas (25), and gastrointestinal stromal tumors (26). Some rare SDH-wt cases have shown that the occurrence of the Carney triad-related gastrointestinal stromal tumors (GISTs) (27–29) or paragangliomas (PGLs) (30) correlated with a decreased mRNA expression of the SDHC subunits. It is reported that SDHC is correlated with increased metastasis-free survival in malignant pheochromocytoma/paraganglioma (31). Additionally, it is found that there is decreased expression of SDHD in gastric cancer (32). Nevertheless, SDH factors are rarely reported in COAD, which indicates that the correlation between SDH factors and COAD remains to be explored.

In this study, we investigated SDHs’ expression in pan-cancer and prediction in the prognosis of COAD patients. Furthermore, the associations among SDHs, immune infiltration, and immunotherapy are explored. To sum up, our results prompted that SDHs may become novel cancer biomarkers in COAD, which act as an immunomodulatory derivative from the tricarboxylic acid cycle, participating in the occurrence and development of COAD.

## Material and methods

### Data collection and variation analysis

Fragments per Kilobase Million (FPKM) normalized expression profile data of pan-cancer, including 33 cancers of The Cancer Genome Atlas (TCGA) database, were downloaded from Genomic Data Commons (GDC) database (https://portal.gdc.cancer.gov/) and merged into an expression matrix. According to human gene annotations (Homo_sapiens.GRCh38.101.CRH.GTF), the Ensemble IDs were transformed into gene symbols. Then, the clinical data of patients with 36 Cholangiocarcinoma (CHOL), 453 Colon adenocarcinoma (COAD), 370 Liver hepatocellular carcinoma (LIHC), 165 Rectum adenocarcinoma (READ), and 370 Stomach adenocarcinoma (STAD) were downloaded and combined into another matrix, respectively. The expression matrix of COAD was stored in **Table S1**, and the clinical characteristics of COAD patients were documented in **Table S2**.

### Expression and prognostic significance of SDHs in COAD

To investigate the difference in gene expression between cancer tissues and normal tissues, we first compared the raw data (Counts) of differentially expressed genes (DEGs) between normal tissues and CHOL, COAD, LIHC, READ, and STAD, respectively, with a threshold of false discovery rate (FDR) < 0.05 by R package “limma”. 168 mitochondrial energy metabolism genes (MMGs) were obtained from KEGG PATHWAY database (https://www.kegg.jp/kegg/pathway.html) (33) and 1476 HIF-1α related genes were downloaded in the GeneCards database (https://www.genecards.org/). After intersecting with MMGs and HIF-1α related genes, a total of 8 DEGs overlapped were recognized. Then, 8 DEGs were used to build the protein-protein interaction (PPI) network by the Search Tool for the Retrieval of Interacting Genes (STRING) 11.0 and visualized in Cytoscape 3.8.2. The expression of 8 DEGs was visualized by R package “pheatmap”. Then, Kaplan-Meier survival analysis, which applied two-sided log-rank tests with a threshold of p < 0.05, was performed on patients with CHOL, COAD, LIHC, READ, and STAD based on 8 DEGs with R package “survminer”. Additionally, a gene expression omnibus (GEO) dataset, GSE14333, which contained the microarray-based of 226 COAD patients and corresponding clinical data, respectively, were downloaded from GEO website.

### Genetic variations of SDHs in COAD

To explore genetic variations of succinate dehydrogenases (SDHs), cBioPortal (http://www.cbioportal.org), a database for cancer genomics data including mutations, and copy number alternations (CNA) from GISTIC, was applied. The mutation profiles of SDHs came from Colorectal Adenocarcinoma (TCGA, PanCancer Atlas) with 526 patients.

### Correlation, functional enrichment based on MMGs

With the RNAseq data of COAD from TCGA, correlation analysis between SDHs and MMGs was visualized by R package “pheatmap”. Additionally, 4 SDHs as well as 168 MMGs were used to build the PPI network by the STRING and visualized in Cytoscape, which involves 41 genes. With the criteria of FDR < 0.05, Gene Ontology (GO) enrichment and Kyoto Encyclopedia of Genes and Genomes (KEGG) pathway analysis were performed utilizing R package “clusterProfiler” based on 41 genes and described by R package “ggplot2”.

### Relationship between the expression of SDHs and the clinical characteristics of patients with COAD

To figure out SDHs’ link with the clinical characteristics of patients with COAD, we analyzed the correlation between the expression levels of SDHs and various clinical characteristics, including T stage, N stage, M stage, age, and lymphatic invasion. Furthermore, Univariate and Multivariate Cox Regression Analysis were conducted to test whether SDHs can be considered independent prognostic factors. R package “rms” and “survival” were employed to formulate a nomogram, which is used to individualize the survival probability for 1-year, 3-year, and 5-year overall survival (OS). Then, time-dependent ROC analysis and Calibration curve were applied to evaluate the nomogram’s discrimination and calibration (34).

### Association between SDHs and immune infiltration

To characterize the immune microenvironment of patients with COAD, based on the expression matrix of SDHs, ssGSEA analysis was performed to visualize the correlation between SDHs and immune infiltration level of 24 immune cell types through R package “GSVA”. Correlation analysis was applied to clarify the SDHs expression in connection with the expressions of immune-related genes. The Tumor Immune Single-Cell Hub (TISCH) database (http://tisch.comp-genomics.org/home/), a scRNA-seq database focusing on the tumor microenvironment, was employed to analyze the correlations between SDHs expression and infiltrating immune cells (35). Gene expression data was gained from the GEO database (GSE146771), including 10468 single cells from 10 patients. The expression of SDHs in different cell types based in GSE146771 was visualized using TISCH.

### Immunotherapy outcomes prediction

The correlation heatmap between SDHs and each immunosuppressive and immunostimulatory gene was visualized by R package “pheatmap”. A total of 18 immunosuppressive genes including ADORA2A, BTLA, CD244, CD274, CD96, CSF1R, CTLA4, HAVCR2, IL10RB, KDR, LAG3, LGALS9, PDCD1, PDCD1LG2, PVRL2, TGFB1, TGFBR1, and TIGIT were selected. A total of 18 MHC molecules including B2M, HLA-A, HLA-B, HLA-C, HLA-DMA, HLA-DOA, HLA-DPA1, HLA-DPB1, HLA-DQA1, HLA-DQA2, HLA-DQB1, HLA-DRA, HLA-DRB1, HLA-E, HLA-F, HLA-G, TAP1, TAP2, and TAPBP were selected. A total of 43 immunostimulatory genes including C10orf54, CD27, CD276, CD28, CD40, CD40LG, CD48, CD70, CD80, CD86, CXCL12, CXCR4, ENTPD1, HHLA2, ICOS, ICOSLG, IL2RA, IL6, IL6R, KLRC1, KLRK1, LTA, MICB, NT5E, PVR, RAET1E, TMEM173, TMIGD2, TNFRSF13B, TNFRSF13C, TNFRSF14, TNFRSF18, TNFRSF25, TNFRSF4, TNFRSF8, TNFRSF9, TNFSF13, TNFSF13B, TNFSF14, TNFSF15, TNFSF4, and TNFSF9 were selected.

The Cancer Immunome Atlas (https://tcia.at/) characterized the intratumoral immune landscapes and the cancer antigenomes from 20 solid cancers. The immunophenoscore (IPS) data of COAD patients was extracted for the following analysis to predict the response to immunotherapy, including the anti-PD-1/PD-L1 treatment and anti-CTLA-4 treatment scores. The microsatellite instability (MSI) was downloaded from cBioPortal and the consensus molecular subtypes (CMS) was obtained from a previous study (36).

### Targeted drug therapy outcomes prediction

To predict targeted drug therapy outcomes according to SHDs’ expression, R package “pRRophetic” was utilized in Axitinib, Cetuximab, GDC0941, and Gefitinib based on the Genomics of Drug Sensitivity in Cancer (GDSC). The natural log of the half-maximal inhibitory concentration (LN_IC50 value) of chemotherapy drugs was downloaded from the GDSC, using GDSC2 screening set. The box plots were drawn by R package “ggplot2”.

### Validation of SDHs at gene and protein levels

The 19 paired COAD tissues were collected from patients who underwent surgical resection for COAD at the Second Affiliated Hospital of Wenzhou Medical University (Wenzhou, China). The corresponding Paraffin section was collected from the Pathology Department of the Second Affiliated Hospital of Wenzhou Medical University (Wenzhou, China). It has passed the examination of the Ethics Committee at Wenzhou Medical University.

The protein expression level of SDHs in COAD and normal tissue was verified by immunohistochemistry (IHC). Sections were dewaxed and rehydrated. The catalase blocker blocked endogenous peroxidase activity (ZSGB-BIO), and the antigen was repaired by sodium citrate buffer (pH 6.0). Then, the tissue sections were incubated overnight with rabbit monoclonal anti-SDHA antibody (1:100 dilution, Proteintech), rabbit monoclonal anti-SDHB antibody (1:100 dilution, Santa cruz), rabbit monoclonal anti-SDHC antibody (1:100 dilution, Proteintech), and rabbit monoclonal anti-SDHD antibody (1:100 dilution, Affbiotech) at 4°C, respectively. After the antibodies were washed, the slices were incubated for 30 minutes with goat anti-rabbit IgG at 37 °C. Then, we redyed with hematoxylin the slices, used neutral gum to seal the shee, and observed it under the optical microscope. Additionally, protein expression of SDHs was downloaded from the Proteomic Data Commons (https://proteomic.datacommons.cancer.gov/pdc/).

The primers of SDHA, SDHB, SDHC, and SDHD can be found in **Table S3**. The total RNA was extracted using TRNzol Reagent and was reverse-transcribed with ReverTra Ace^®^qPCR RT Master Mix with gDNA Remover (TOYOBO, Japan). All qPCR reactions were performed with Hieff^®^ Qpcr SYBR Green Master Mix(Yeasen Biotechnology (Shanghai)) in 20µl volume containing 10µl 2× SYBR Green RT-PCR Master Mix, 0.4µl of each 0.2µM forward and reverse primer, 1µl of cDNA sample, and nuclease-free water up to 20µl. Amplification was carried out according to the following conditions: initial denaturation at 95°C for 5 min, followed by 40 cycles of denaturation at 95°C for 10s, and annealing at 60°C for 30s. The relative expression of the gene was calculated by the 2^-△Ct method.

## Results

### Expression and prognostic significance of SDHs in COAD

**Figure 1:**
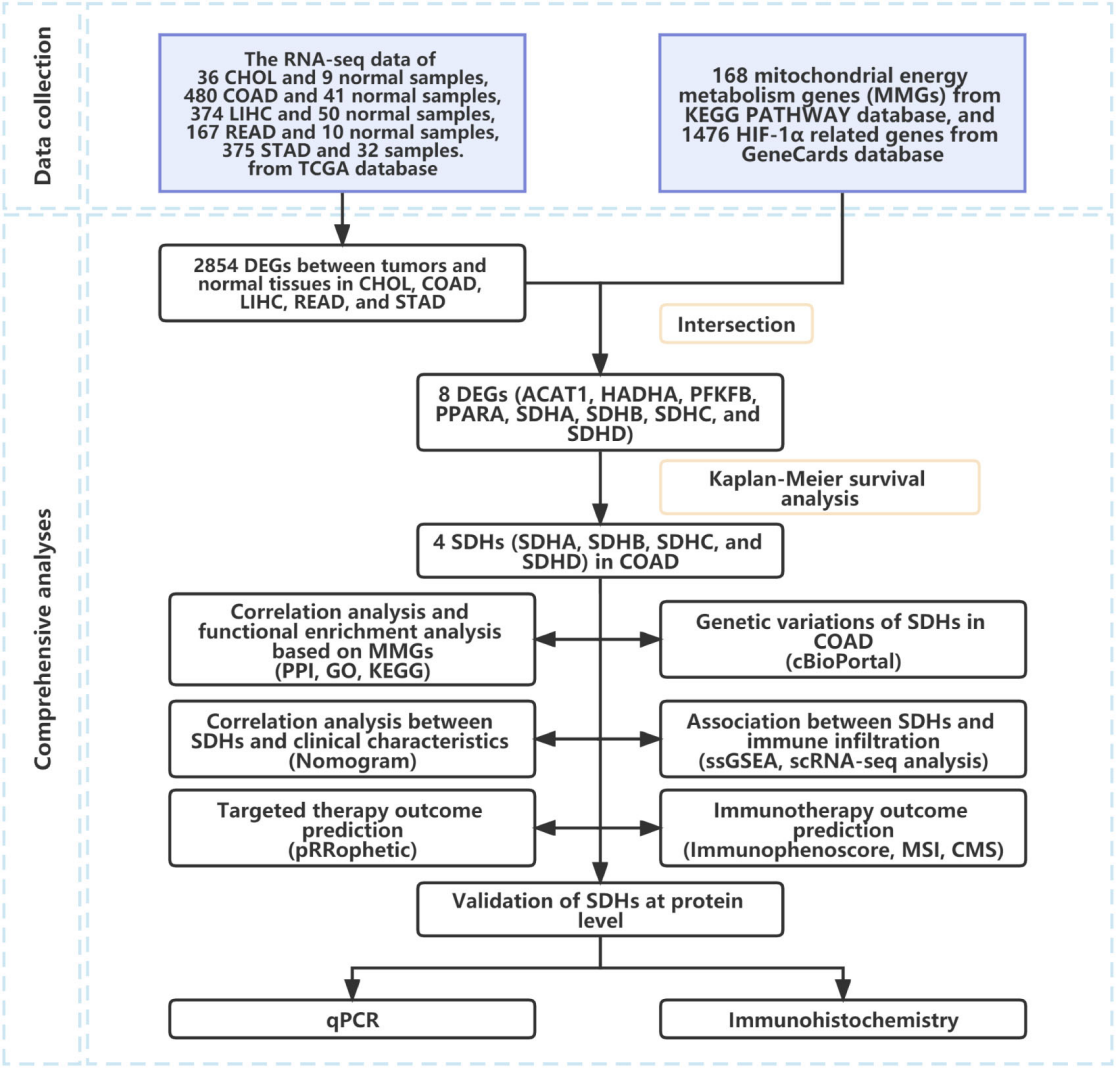
Flowchart of the study process.

The workflow of our study was shown in **Figure 1**.

**Figure 2:**
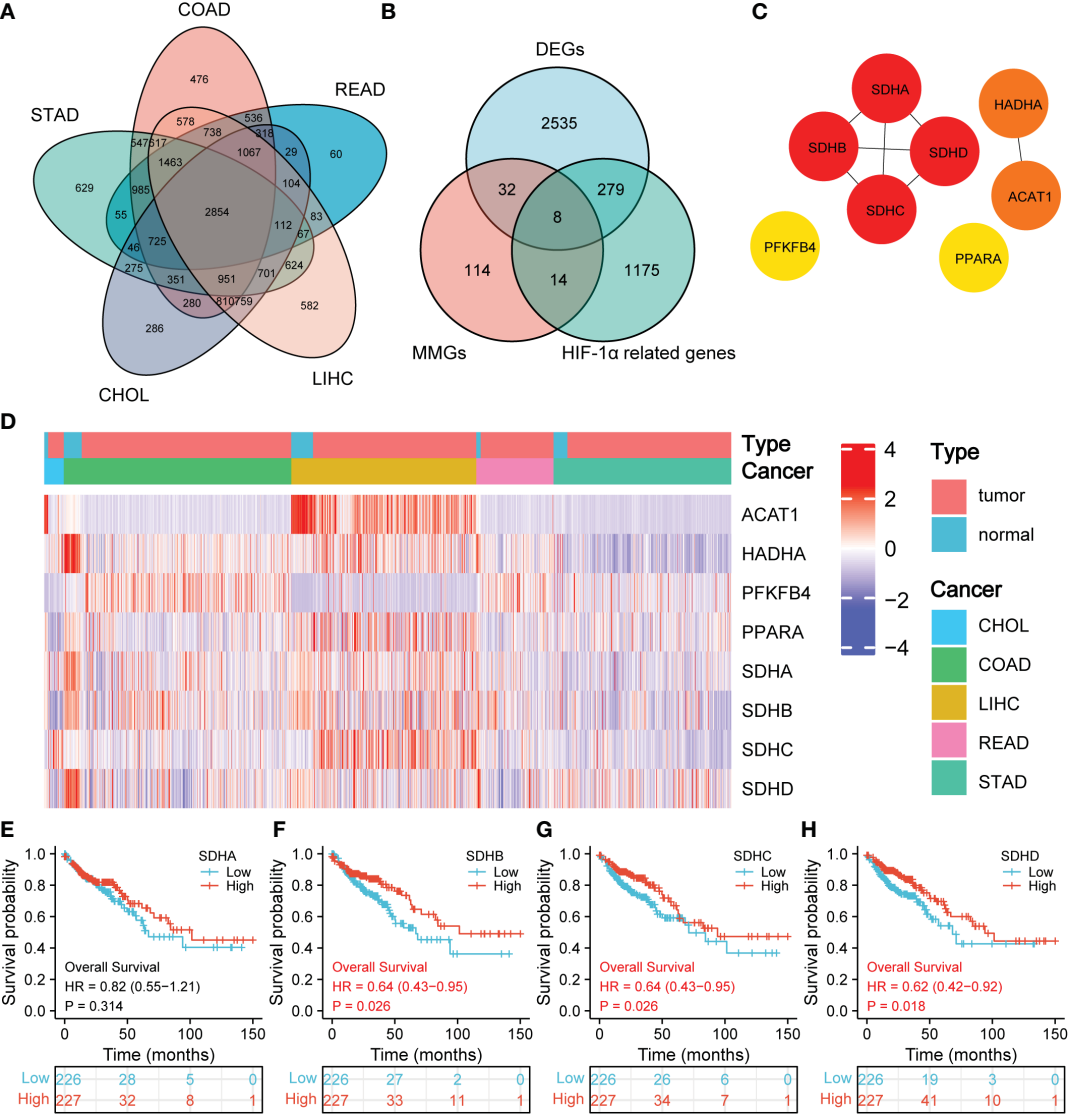
Prognostic significance of SDHs. **(A)** Venn diagram of DEGs in CHOL, COAD, LIHC, READ, and STAD. **(B)** Venn diagram of DEGs, MMGs, and HIF-1α-related genes. **(C)** The network for 8 DEGs intersected. **(D)** Heatmap of SDHA, SDHB, SDHC, and SDHD between 41 normal tissues and 453 COAD patients. **(E-H)** Kaplan-Meier overall survival of SDHA, SDHB, SDHC, and SDHD in COAD.

We first compared the raw data (Counts) of differentially expressed genes (DEGs) between normal tissues and CHOL, COAD, LIHC, READ, and STAD, respectively, with a threshold of false discovery rate (FDR) < 0.05. Ultimately, a total of 2854 DEGs were identified (**Figure 2A**). Then, the intersection of 2854 DEGs, 168 mitochondrial energy metabolism genes (MMGs), and 1476 HIF-1α related genes included 8 DEGs (ACAT1, HADHA, PFKFB, PPARA, SDHA, SDHB, SDHC, and SDHD) (**Figure 2B**). 168 MMGs were obtained from KEGG PATHWAY database, and 1476 HIF-1α related genes were downloaded from GeneCards database. The DEGs, MMGs, and HIF-1α-related genes were listed in **Table S4**. In addition, a protein-protein interaction (PPI) network with 8 DEGs was constructed through the Search Tool for the Retrieval of Interacting Genes (STRING) (**Figure 2C**, **Table S5**). According to the PPI network, there exists a strong relationship among SDHA, SDHB, SDHC, and SDHD. After reviewing the literature, we found that SDHA, SDHB, SDHC, and SDHD belong to the family of succinate dehydrogenase (SDH) (16).

**Figure 2D** demonstrates the expression of 8 DEGs in CHOL, COAD, LIHC, READ, and STAD between normal tissues and pathological tissues, and the volcano figures were stored in **Supplementary Figure 1**. Especially for SDHs in COAD, the expression of SDHA, SDHB, SDHC, and SDHD in normal tissues is higher than that in COAD patients. To have a comprehensive insight into the prognostic value of 8 DEGs, Kaplan-Meier survival analysis was applied to patients with CHOL, COAD, LIHC, READ, and STAD. The results were shown in **Supplementary Figure 2** and **3**. It’s revealed that SDHB (p = 0.026), SDHC (p = 0.026), and SDHD (p = 0.018) were significantly associated with the prognosis of COAD in **Figures 2E–H**. The survival time in the high expression group of SDHB, SDHC, and SDHD was longer than that in the low expression group, which indicates that high expression of SDHB, SDHC, and SDHD benefits COAD patients. Additionally, the consistent results obtained from GSE14333 make the conclusion more convincing (**Supplementary Figure 4**).

### Genetic variations of SDHs in COAD

**Figure 3:**
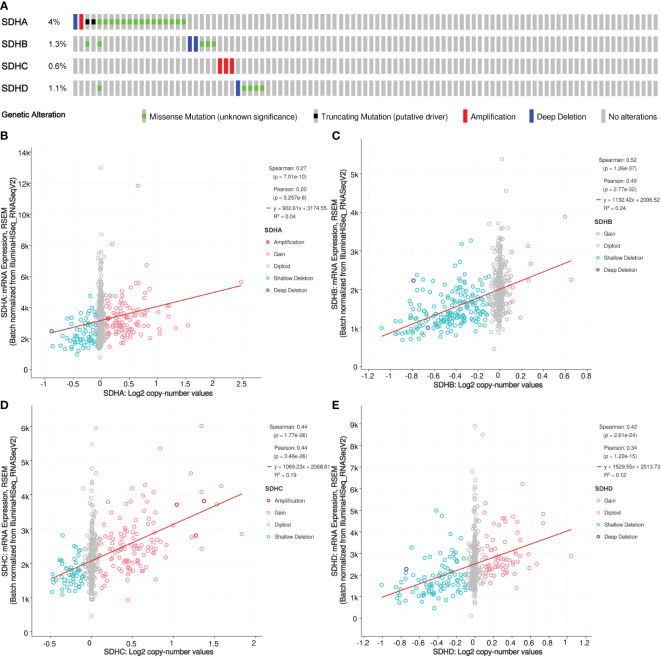
Somatic mutation of SDHs. **(A)** Genetic mutation analysis of SDHs. **(B-E)** Relationship between CNA in SDHs and expression of mRNA.

To explore genetic variations of SDHs, cBioPortal was applied. The mutation profiles of SDHs came from Colorectal Adenocarcinoma (TCGA, PanCancer Atlas) with 526 patients. As shown in **Figure 3A**, a high mutation rate of SDHs was observed in COAD patients. Among all SDHs, SDHA is regarded as the top gene in mutation frequency rank in COAD patients (4%). Furthermore, the correlation between SDHs copy number alternations (CNA) and expression of mRNA was presented in **Figures 3B–E**, pointing out that a positive correlation was found between SDHs copy number and mRNA expression in COAD.

### Correlation, functional enrichment based on MMGs

**Figure 4:**
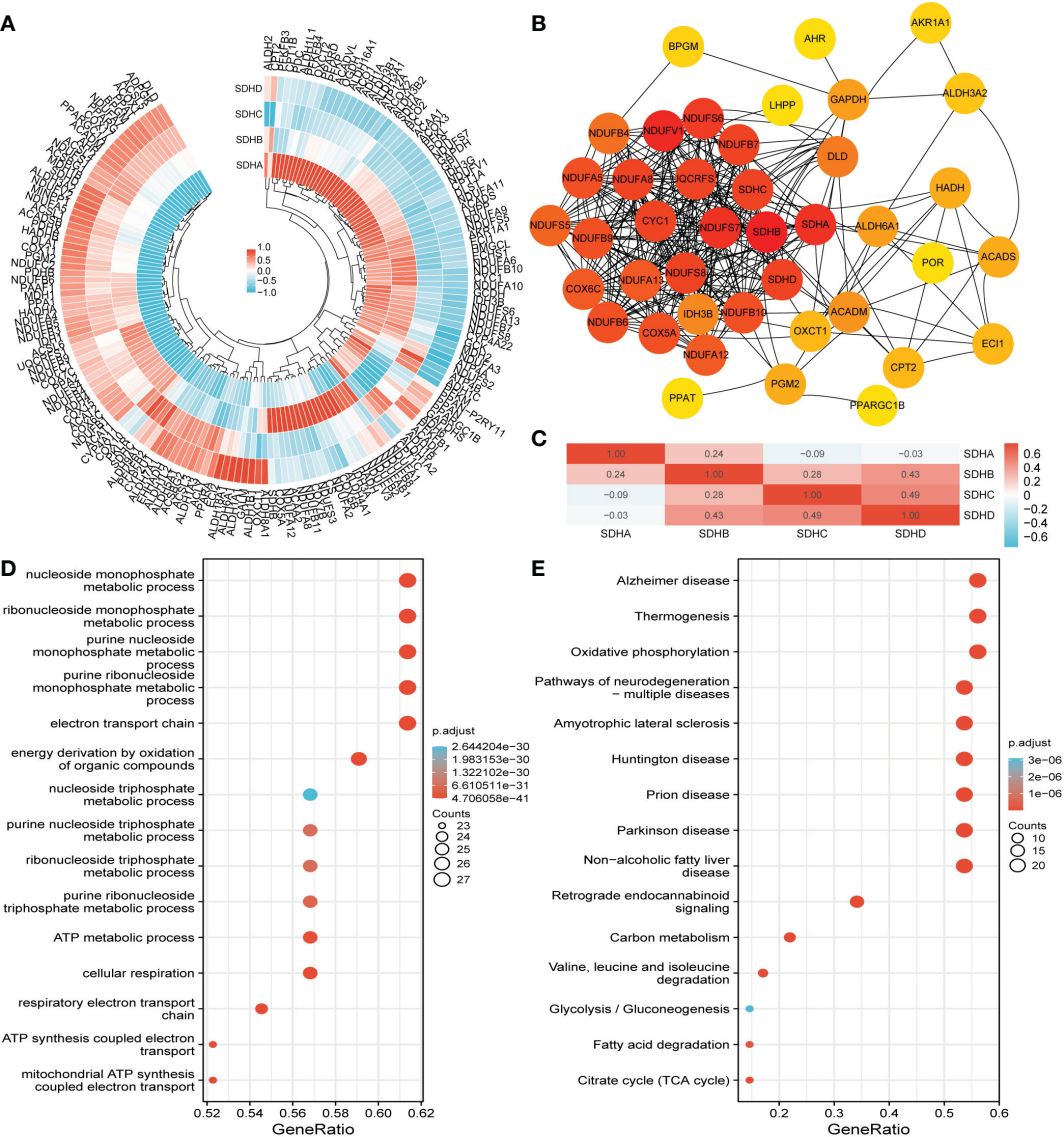
Correlation, functional enrichment based on MMGs. **(A)** The correlation between MMGs and SDHs in COAD. **(B)** The network for 41 genes is based on SDHs and MMGs with the highest correlation. **(C)** The correlation between different SDHs in COAD. **(D, E)** The functions and pathways based on 41 genes were predicted by the analysis of GO and KEGG.

Considering that SDHs have a strong connection with energy metabolism, correlation analysis was conducted between SDHs and MMGs. The results were shown in **Figure 4A**, and the detailed data was demonstrated in **Table S6**, indicating the significant correlations between 4 SDHs and 168 MMGs. In addition, a PPI network with 4 SDHs as well as 168 MMGs was constructed through the STRING, involving 41 elements (**Figure 4B**, **Table S7**). Additionally, a correlation analysis between 4 SDHs was shown in **Figure 4C**, reflecting that SDHs have a strong correlation except for SDHA. Furthermore, Gene Ontology (GO) enrichment and Kyoto Encyclopedia of Genes and Genomes (KEGG) pathway analysis were performed to predict the functions and pathways of 41 genes (**Table S8**). It is demonstrated that these SDHs-related genes involve electron transport chain, respiratory electron transport chain, mitochondrial ATP synthesis coupled electron transport, and respiratory chain complex in GO enrichment analysis (**Figure 4D**). Additionally, according to KEGG analysis, it is mirrored that these genes are relative to oxidative phosphorylation (OXPHOS), citrate cycle (TCA cycle), and glycolysis/Gluconeogenesis (**Figure 4E**). The results showed that SDHs-related genes were enriched in electron transport chain, respiratory electron transport chain, cellular respiration, respiratory chain, oxidative phosphorylation, carbon metabolism, and TCA cycle.

### Relationship between the expression of SDHs and the clinical characteristics of patients with COAD

**Figure 5:**
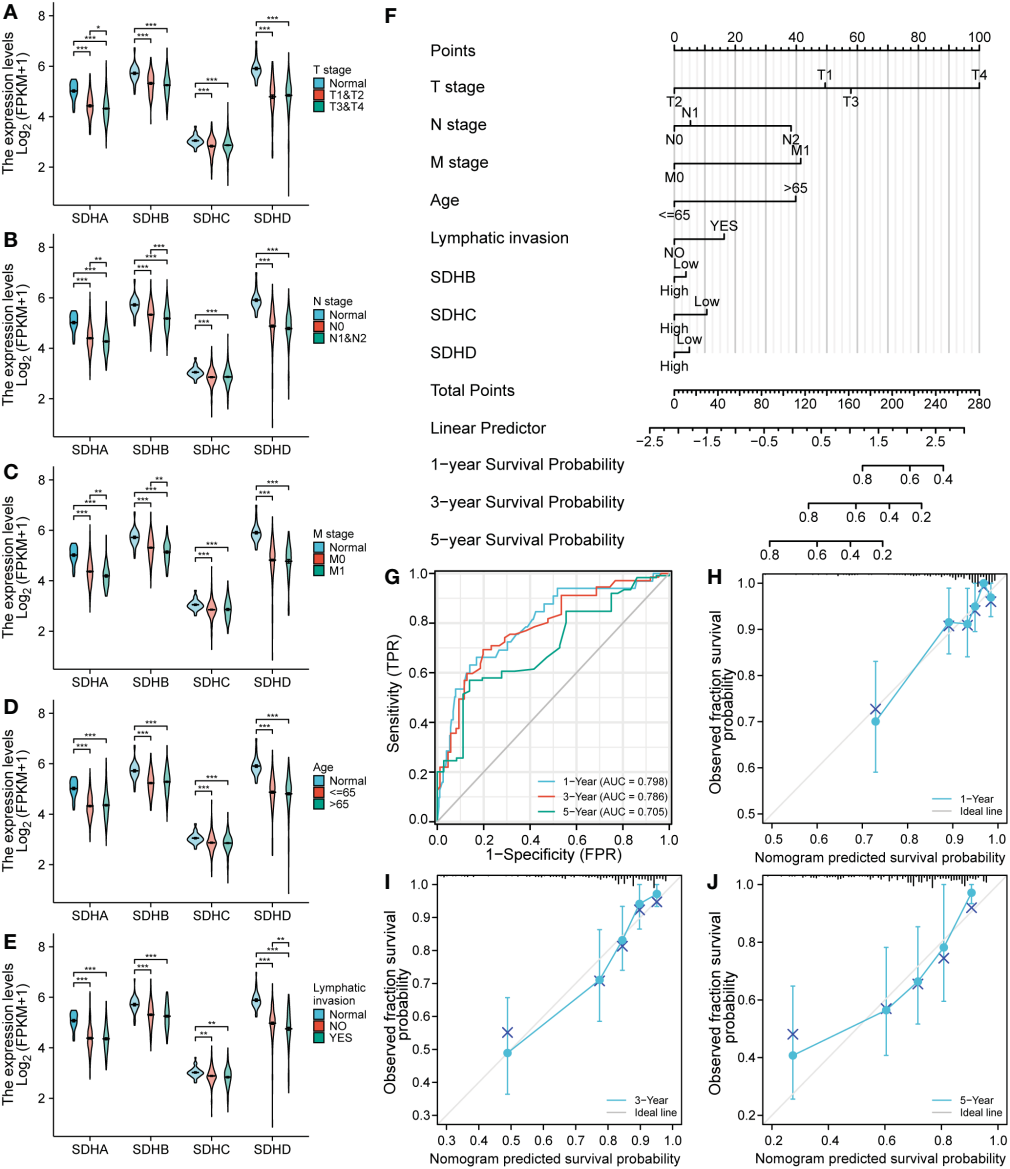
SDH factor expression and the clinical characteristics of patients with COAD. **(A-E)** SDHA, SDHB, SDHC, and SDHD are concerned with clinical characteristics involving T stage, N stage, M stage, age, and lymphatic invasion. **(F)** A nomogram to predict the overall survival rate of COAD patients. **(G-J)** Time-dependent ROC analysis and Calibration curve for the overall survival nomogram model in the discovery group. A dashed diagonal line represents the ideal nomogram. *p < 0.05; **p < 0.01 and ***p < 0.001; ns, not significant.

To figure out the connection between SDHs and the clinical characteristics of patients with COAD, including T stage, N stage, M stage, age, and lymphatic invasion, the violin diagram were drawn in **Figures 5A–E**. To illustrate, the T stage, N stage, and M stage represent the extent of primary cancer, the regional lymph node involvement, and the distant metastasis, respectively, based on evidence obtained from clinical assessment parameters determined prior to treatment. Additionally, Lymphatic invasion is a yes/no indicator to ask if malignant cells are present in small or thin-walled vessels suggesting lymphatic involvement. It’s pointed out that SDHA and SDHB were low expressed in higher N and M stages (**Figures 5B, C**). There exists no positive result in age (**Figure 5D**), however, it is demonstrated that SDHD expression levels were all lower in lymphatic invasion samples (**Figure 5E**). Summarily, there is a close relationship between SDHs and clinical characteristics.

To testify whether SDHs can be regarded as independent prognostic factors, Univariate and Multivariate Cox Regression Analysis were employed in COAD patients. The results were demonstrated in **Table 1**, and the risk score of the nomogram and the coefficient of clinical characteristics were documented in **Table S9**. It’s revealed that SDHB, SDHC, SDHD, and clinical characteristics involving T stage, N stage, M stage, age, and lymphatic invasion were correlated with the survival of COAD patients. A nomogram is formulated based on independent prognostic factors to predict the survival probability individually (**Figure 5F**). For each COAD patient,1-, 3-, and 5-year survival rates would be predicted by the total points in the nomogram accor to 8 indicators. To assess the sensitivity and specificity of this nomogram, time-dependent receiver operating characteristic (ROC) analysis was adopted. The ROC area under the curve (AUC) is 0.798 for 1-year, 0.780 for 3-year, and 0.705 for 5-year survival, representing an efficient predictive efficacy (**Figure 5G**). Then, the Calibration curve was applied to evaluate the nomogram’s discrimination and calibration, reflecting an ideal capacity of the nomogram for effectively predicting the prognosis of COAD patients (**Figures 5H–J**).

**Table 1:** Univariate and multivariate Cox regression analysis of SDHs for COAD with clinical characteristics in TCGA cohort.

Characteristics	Total (N)	Univariate analysis	
		Hazard ratio (95% CI)	P value
**T stage**	452		
T1	11	Reference	
T2	77	0.453 (0.088-2.347)	0.346
T3	308	1.326 (0.325-5.409)	0.694
T4	56	3.826 (0.893-16.394)	0.071
**N stage**	453		
N0	266	Reference	
N1	105	1.635 (0.991-2.695)	0.054
N2	82	3.997 (2.549-6.266)	**<0.001**
**M stage**	396		
M0	332	Reference	
M1	64	4.327 (2.763-6.776)	**<0.001**
**Age**	453		
<=65	188	Reference	
>65	265	1.649 (1.077-2.526)	**0.021**
**Lymphatic invasion**	410		
NO	247	Reference	
YES	163	2.315 (1.520-3.525)	**<0.001**
**SDHA**	453		
Low	226	Reference	
High	227	0.818 (0.554-1.209)	0.314
**SDHB**	453		
Low	226	Reference	
High	227	0.637 (0.429-0.948)	**0.026**
**SDHC**	453		
Low	226	Reference	
High	227	0.640 (0.432-0.947)	**0.026**
**SDHD**	453		
Low	226	Reference	
High	227	0.620 (0.418-0.921)	**0.018**
High	227	1.207 (0.816-1.786)	0.345

The bold letters in the first column represent different clinical characteristics. And the bold letters in the fourth column represent the clinical characteristics is significant (P < 0.05).

### Association between SDHs and immune infiltration

**Figure 6:**
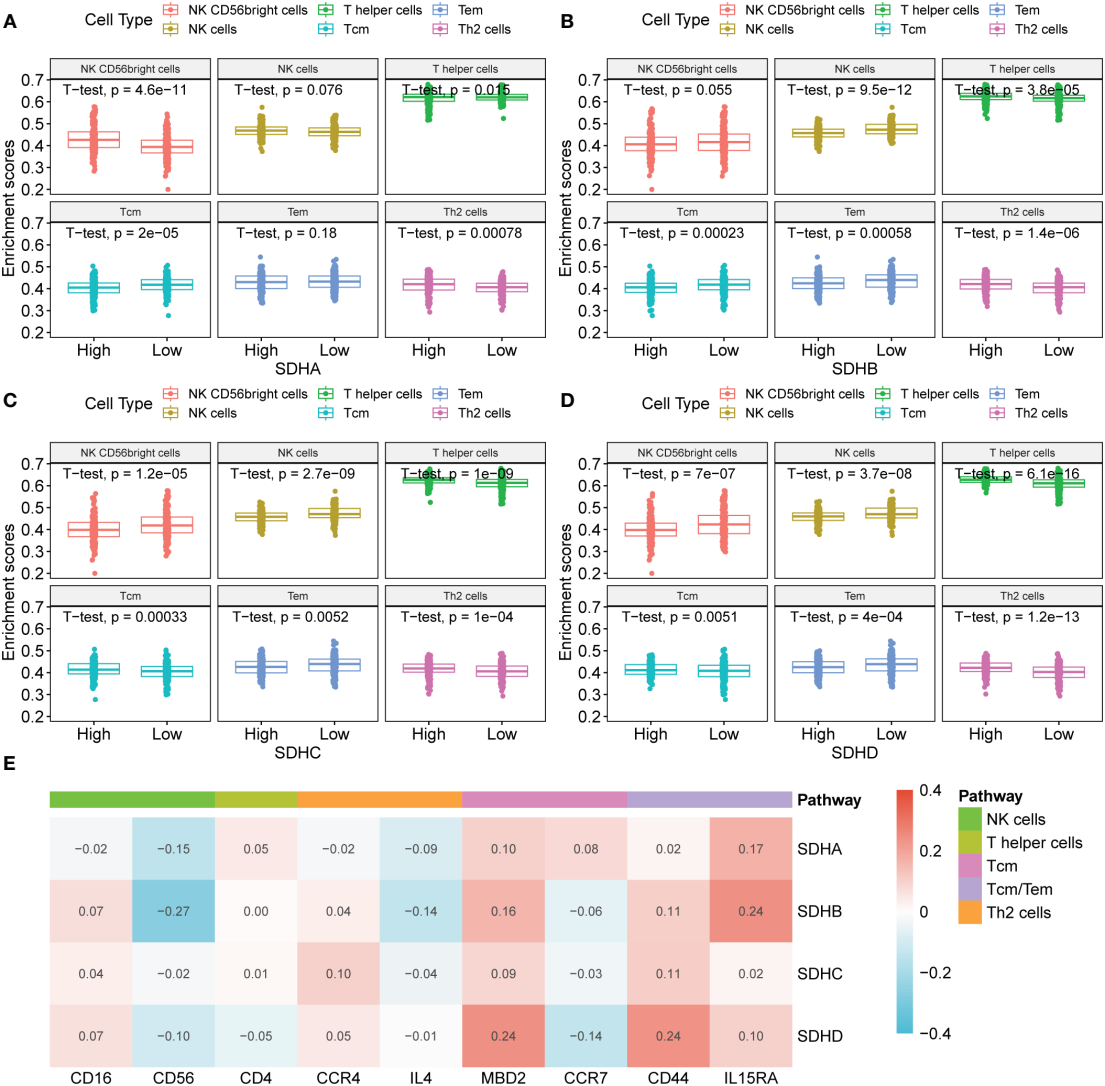
Association between SDHs and immune infiltration. **(A-D)** The ssGSEA analysis based on SDH factor expression for COAD and different types of immune cells. **(E)** The correlations between the SDHs expression and the expressions of mark genes of immune cells.

To investigate the connection between SDHs and immune cells, the ssGESA analysis was performed (**Supplementary Figures 5A-D**, **Table S10**). Among 24 immune cells, there exists a close relationship between SDHs and Tem (Effective Memory T Cell), Tcm (Central Memory T cell), T helper cells, Th2 (T helper 2) cells, NK CD56bright cells, and NK cells (**Figures 6A–D**). Surprisingly, for T helper cells and Th2 cells, SDHs were significantly enriched in the high expression group. As for Tem, SDHs were significantly enriched in the low expression group. Additionally, for Tcm, SDHA and SDHB were significantly enriched in the low expression group, while SDHC and SDHD were enriched in the high expression group. For NK cells, SDHs were significantly enriched in the low expression group except for SHDA. However, SDHA was significantly enriched in the high expression group, while SDHC and SDHD were enriched in the low expression group for NK CD56bright cells. We also examined the correlations between the SDHs expression and the expressions of mark genes of immune cells in **Figure 6E**. It’s indicated that SDHs, especially SDHB, were negatively related to CD56, which is the marker gene of NK cells. In addition, there exists a strong correlation between SDHD and MBD2, a marker gene of Tcm. For CD44 and IL15RA, two genes related to Tcm and Tem, all SDHs are positively correlated with them, especially SDHA, SDHB, and SDHD. To figure out SDHs’ expression in different immune cell types, we analyzed single-cell sequencing datasets of GSE146771 from the Tumor Immune Single-Cell Hub (TISCH) database. In **Supplementary Figure 6**, GSE146771 was divided into 13 cell types. Focusing on the lower left corner of UMAP plots, we can see that SDHs mainly enriched in CD4Tconv cells, CD8T cells, CD8Tex cells, Treg cells, Tprolif cells, and NK cells, which is consistent with ssGSEA results.

### Immunotherapy outcomes prediction

**Figure 7:**
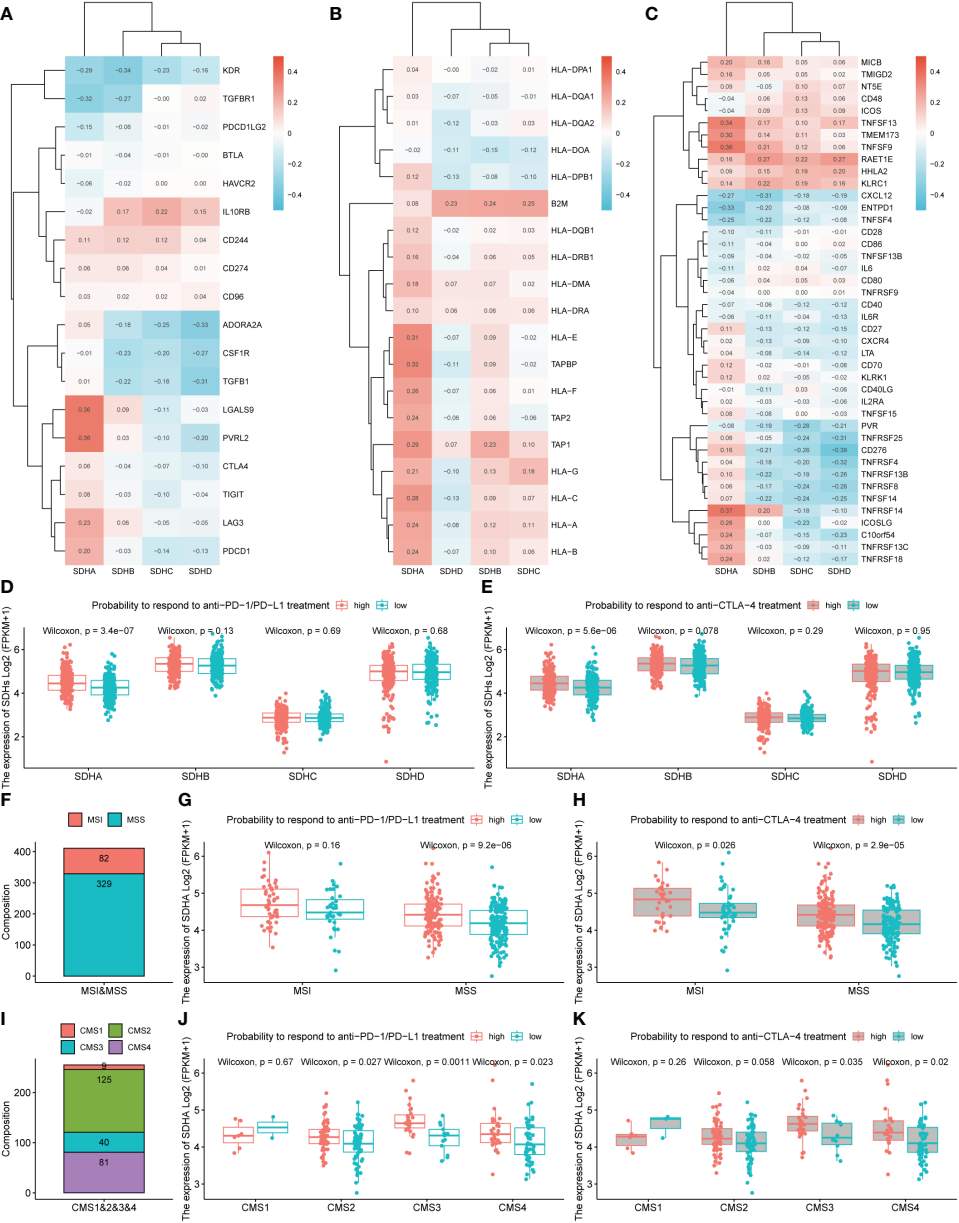
Immunotherapy outcomes prediction. **(A-C)** The correlation between SDHs and immunostimulatory, MHC, and immunosuppressive genes. **(D, E)** The association between SDHs expression and the relative probabilities of responding to immunotherapy, including anti-PD-1/PD-L1 therapy and anti-CTLA-4 therapy. **(F)** Proportions of MSI and MSS in patients. **(G, H)** The possibility to respond to immunotherapy based on different SDHA expression and MSI. **(I)** Proportions of CMS1, CMS2, CMS3, and CMS4 in patients in MSS. **(J, K)** The possibility to respond to immunotherapy in patients in MSS based on different SDHA expression and CMS.

To deepen the understanding of the value of SDHs for COAD treatment, the relationships between SDHs and marker genes of immunostimulation, MHC, and immunosuppression were listed in **Figures 7A–C** and **Table S11-S13**, respectively. It turns out that SDHs are significantly correlated with these immune-related genes. Unlike other SDHs, SDHA had different correlations with immune genes. Interestingly, some immunosuppressants showed uniform correlations. The results indicated that ADORA2A, CSF1R, CTLA4, KDR, PDCD1LG2, TGFBR1, TGFB1, and CXCL12 had significant negative correlations with SDHs while CD244, IL10RB, KLRC1, and RAET1E had significant positive correlations with SDHs. As for genes of MHC, SDHA was positively correlated with almost all genes, especially HLA-E and TAPBP. IPS is a machine learning-based scoring system that could predict patients’ responses to immunotherapy, including anti-PD-1/PD-L1 and anti-CTLA-4 treatment (37). Combined analysis of the expression SDHs and IPS score proved that COAD patients with high SDHA expression are more suitable for immunotherapy such as anti-PD-1/PD-L1 (p = 3.4×10^-7^) and anti-CTLA-4 (p = 5.6×10^-6^) treatment (**Figures 7D, E**, **Table S14**). Furthermore, we explored how the microsatellite instability (MSI) and consensus molecular subtypes (CMS) effect the patients’ possibility to respond to immunotherapy with different SDHA expression. Microsatellite instability (MSI) distribution of patients was displayed in **Figure 7F**. Specifically, patients in microsatellite stability (MSS) with high SDHA expression are more suitable for immunotherapy such as anti-PD-1/PD-L1 (p = 9.2×10^-6^) and anti-CTLA-4 (p = 2.9×10^-5^) treatment (**Figures 7G, H**). Then, we explored how different CMS effect the possibility to respond immunotherapy in patients in MSS with different SDHA expression. Proportions of CMS in patients in MSS were demonstrated in **Figure 7I**. Patients in CMS3 and CMS4 with high SDHA expression have a higher possibility to respond to immunotherapy (**Figures 7J, K**).

### Targeted drug therapy outcomes prediction

**Figure 8:**
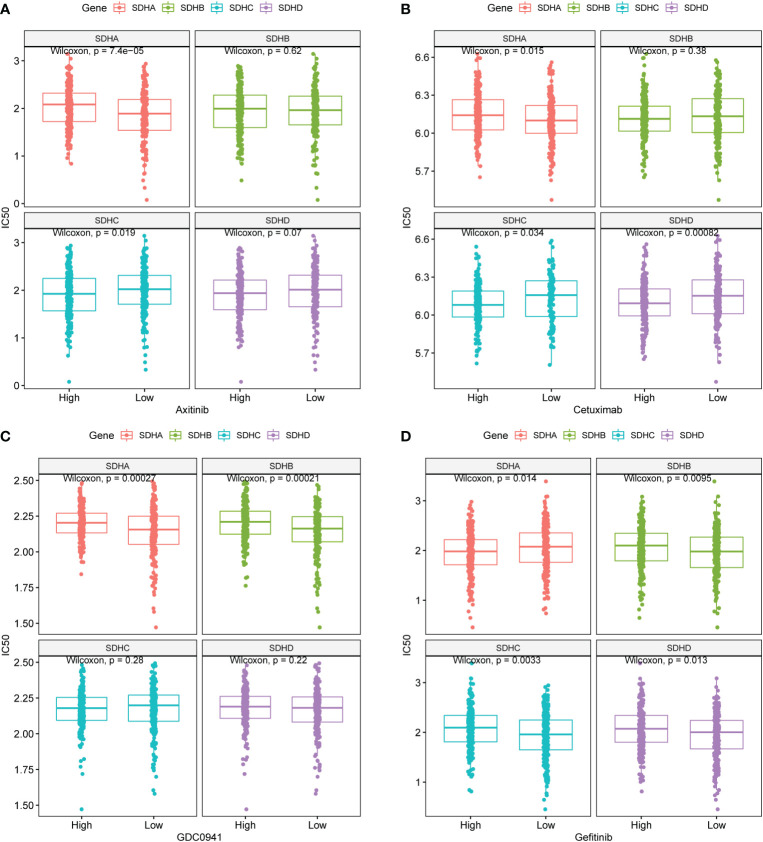
Targeted drug therapy outcomes prediction. **(A-D)** GDSC predicts the IC50 difference of four drugs between COAD patients with different SDHs expression.

To investigate the relationship between SDHs and targeted drug sensitivity, the Genomics of Drug Sensitivity in Cancer (GDSC) of Axitinib, Cetuximab, GDC0941, and Gefitinib was utilized. The result indicated significant differences in targeted drug sensitivity in COAD patients with different SDHs’ expression (**Figures 8A–D**, **Table S15**). Specifically, COAD patients with different SDHA expression have different responses to Axitinib, Cetuximab, GDC0941, and Gefitinib. Additionally, with higher SDHB expression, COAD patients are more sensitive to GDC0941 and Gefitinib. However, the drug sensitivity of COAD patients with high expression of SDHC and SDHD is opposite to that of COAD patients with high expression of SDHA.

### Validation of SDHs at gene and protein levels

To validate the consistency between the gene level and protein level of SDHs in COAD, we evaluated the protein expressions of SDHs in COAD through Proteomic Data Commons (PDC) database and Immunohistochemistry (IHC).

**Figure 9:**
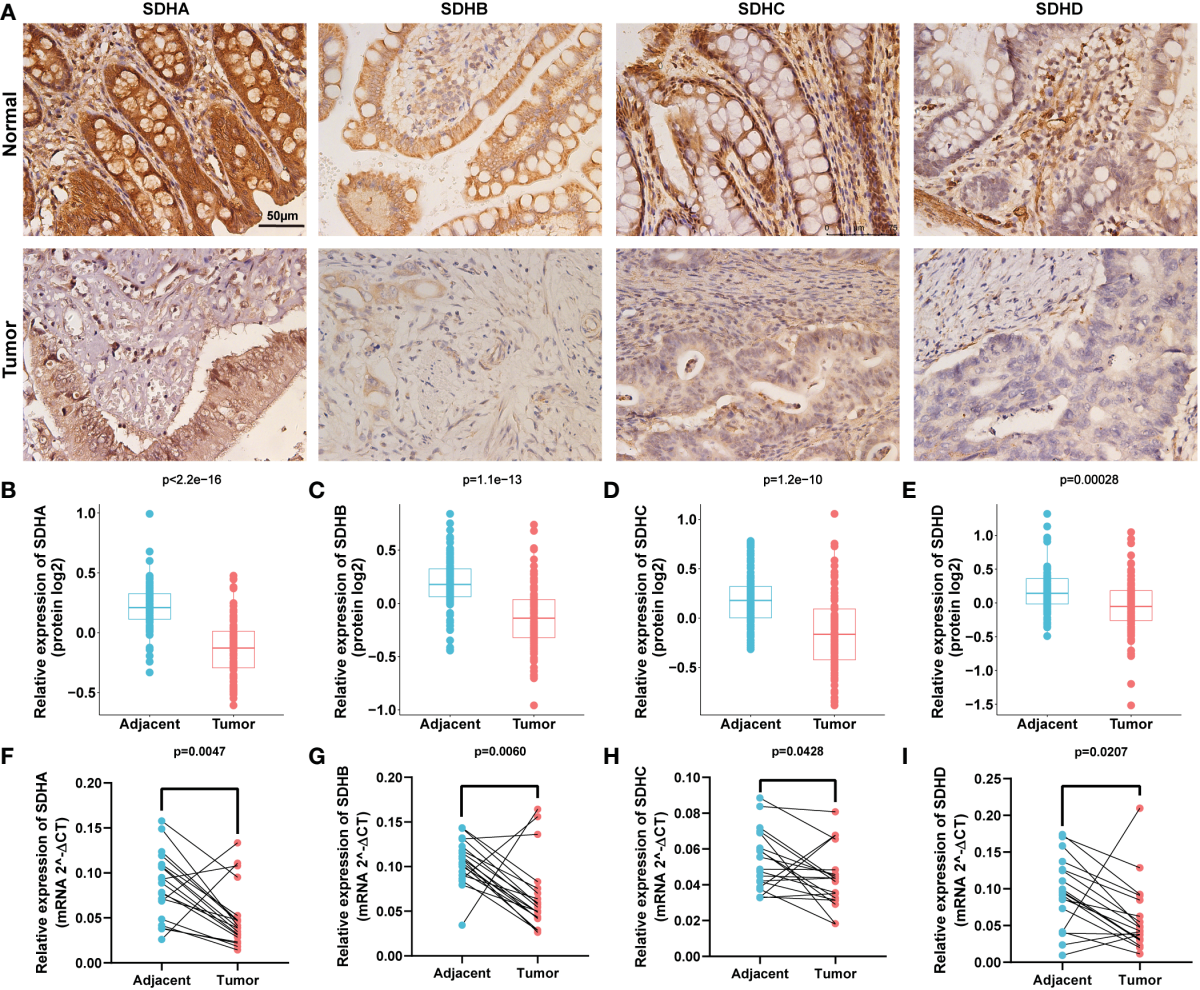
Validation of SDHs at gene and protein levels. **(A)** IHC of SDHs in COAD and normal tissues. **(B-E)** The protein expression of SDHs in COAD in Proteomic Data Commons database. **(F-I)** The relative mRNA expression level of SDHs in COAD and adjacent normal tissues detected by qPCR.

**Figure 9A** indicated that SHDs, mainly located in the cytoplasm, were mainly expressed in glandular cells. Furthermore, the immunohistochemical staining intensity of SDHA, SDHB, SDHC, and SDHD in normal tissues was more substantial than in COAD tissues, demonstrating that these proteins were more significantly expressed in adjacent colon tissues than in COAD tissues. According to the PDC database, compared with normal tissues, SDHA (**Figure 9B**, p < 2.2×10^-16^), SDHB (**Figure 9C**, p = 1.1×10^-13^), SDHC (**Figure 9D**, p = 1.2 ×10^-10^), and SDHD (**Figure 9E**, p = 0.00028) were low expressed in colon cancer at protein level.

In addition, qPCR with 19 paired tumors and adjacent tissues was performed, suggesting that the mRNA expression of SDHs was significantly different from tumors and adjacent tissues (**Figures 9F–I**). These results showed that all SDHs have good consistency between gene and protein levels, which was highly expressed in colon tissues and low expressed in colon cancer.

## Discussion

A growing body of research proved mitochondrial metabolism plays an essential role in tumorigenesis, metastasis, and treatment resistance (7, 38–42). Succinate dehydrogenase (SDH), a tumor metabolite, acts as an oncogenic signaling molecule in many cellular processes such as metabolic and epigenetic alterations, angiogenic stimulation, migration, invasion, and post-translational modification of proteins (43). Consequently, we found that high expression of SDHB, SDHC, and SDHD has a better prognosis for COAD patients, reflecting that all of them can be defined as protective factors for COAD by TCGA and GSE14333 data analysis.

Mutations in genes are known to be closely linked to the development of malignant tumors. The mutation of SDH in the development and prognosis of several cancers has been partially established (26–30, 44). In Carney triad (CT) patients, a high methylation level of SDHC was found, which was correlated to functional impairment of the SDH complex (29). And the notable immunohistochemical loss of SDHA in gastrointestinal stromal tumors (GISTs) signals mutation of SDHA (45). Therefore, we explored the genetic variations of SDHs in COAD through cBioPorta. For SDHs, mutations are positively correlated with mRNA expression. Interestingly, it’s found that in COAD, SDHA is the top gene in mutation frequency rank and is mainly involved in the missense mutation in COAD. COAD progression can be hindered by inhibiting mitochondrial OXPHOS through Lin28a/SDHA signaling pathway (46). Additionally, SDHA inactivation results in the accumulation of succinate, which binds to and activates thioredoxin reductase 2, a reactive oxygen species-scavenging enzyme, to render chemotherapy resistance in COAD (47). Therefore, we speculate that SDHA may be involved in the progression and treatment of COAD as a critical gene among the SDHs.

To further explore the link between SDHs and energy metabolism in COAD, the correlation analysis and PPI between SDHs and MMGs were conducted, indicating the significant correlations between SDHs and MMGs. Additionally, the correlation analysis between 4 SDHs reflected that SDHs have a strong correlation except for SDHA. Meanwhile, we found that SDHs-related genes were enriched in electron transport chain, OXPHOS, carbon metabolism, and TCA cycle by correlation analysis and functional enrichment analysis. It’s important to note that previous studies have shown that the TCA cycle and carbon metabolism have a particular impact on the prognosis of patients with COAD (48, 49). It has been reported that SDHB gene knockout in the human pheochromocytoma cell line (HPheo1) up-regulates genes involved in glycolysis and down-regulates genes involved in OXPHOS (50). Glycolylysis-dependent impaired OXPHOS has also been shown in familial renal cancer patients with germline mutations of the SDHB gene (51). Our analysis revealed that SDHs play a role in the TCA cycle and metabolism process pathway. In terms of tumor metabolism, the glycolysis/oxidative phosphorylation (OXPHOS) ratio is of great significance in tumorigenesis.

In recent years, cancer immunotherapy has generally drawn the public’s attention, which was named 2013’s Breakthrough of the Year by Science (52). Up to now, checkpoint inhibitors have been the most thoroughly investigated class of immunotherapy. So far, five PD-1 or PD-L1 inhibitors and one CTLA4 inhibitor have been approved to treat various cancers based on improvements in overall survival (53). However, many patients do not respond to treatment with checkpoint inhibitors. The factors underlying responsiveness to checkpoint inhibitors are being intensely studied (54). When activated, T cells express programmed cell death 1 (PD-1) for recognizing abnormal and cancerous cells (55, 56). cytotoxic T lymphocyte antigen 4 (CTLA4), is a co-inhibitory molecule that regulates the extent of T cell activation. blocks the interaction between CTLA4 and these ligands, CD80 and CD86, and keeps T cells remain active, which can recognize and kill tumor cells (57). It has been reported that succinic acid plays a role in the cancer microenvironment and regulates many metabolic pathways through G protein-coupled receptors (58). It is thus clear that as an essential intermediate product of the tricarboxylic acid (TCA) cycle, succinate and SDHs extend beyond metabolism and enter anticancer immunity (59).

To investigate the connection between SDHs and immune infiltration, we explored the association between SDHs and immune infiltration. In our study, the degree of immune infiltration of T helper cells was closely related to the expression of SDHs, which may be caused by the enrichment of SDH in T helper cells leading to enhancement of mitochondrial activity. It’s known that T helper cells are essential for protective immunity and play a role in inflammatory responses to self-antigens or nonharmful allergens (60). Metabolic inhibition decreased T-cell proliferation and activation or led to T-cell anergy or cell death (61–63). Moreover, the low expression level of SDHs was correlated to functional impairment of the SDH complex because of the Warburg effect (64). Nevertheless, the specific function of the Warburg effect in activated T cells remains unclear (65). The functional mechanism of energy metabolism of SDHs on COAD needs to be further explored.

SDHB, SDHC, and SDHD showed high similarity in our correlation analysis between SDHs and marker genes of immunosuppression and immunostimulation. SDHA is regarded as a new target to mitigate T cell-mediated intestinal diseases including alloimmune gastrointestinal graft versus host disease (GI-GVHD), autoimmune inflammatory bowel disease (IBD), and iatrogenic CTLA-4Ig ICB-mediated colitis (66) because this reduction in SDHA caused an enhanced sensitivity of the intestinal epithelial cells (IECs) to T cell-mediated cytotoxicity (67, 68). Our analysis proved that SDHA, positively correlated with most of these gene signatures, has a peculiar pattern regarding gene signatures compared to other SDHs. Additionally, our results indicated that SDHA is significantly associated with Lymphocyte activation gene 3 protein (LAG3), which provides a new direction for immunotherapy in patients with COAD. Highly correlated with LAG3, adoptive cell therapy using tumor-infiltrating lymphocytes (TILs) was a promising immunotherapy approach for COAD (69). Through immunophenoscore (IPS), COAD patients with high SDHA expression are more suitable for immunotherapy such as anti-PD-1/PD-L1 and anti-CTLA-4 treatment. In fact, it’s known that the majority of COAD patients in microsatellite instability (MSS) were less sensitive to immune checkpoint inhibitors than the minority of COAD patients in microsatellite instability (MSI) (70). In our study, MSS patients with different SDHA expression have different possibility to respond to immunotherapy. With high SDHA expression, MSS patients can benefit more from immunotherapy. Consensus molecular subtypes (CMS) groups CRC samples according to their gene-signature in four subtypes: CMS1 (MSI Immune), CMS2 (Canonical), CMS3 (Metabolic), and CMS4 (Mesenchymal) (36). Patients in CMS3 are demonstrated enrichment for multiple metabolism signatures, while patients in CMS4 are likely to be diagnosed at more advanced stages and have poor survival (71). For both CMS3 and CMS4 patients in MSS, higher SDHA expression was associated with better treatment outcomes, indicating that SDHA might become a new biomarker for predicting the outcomes of immune checkpoint blockades such as anti-PD-1/PD-L1 and anti-CTLA-4.

Target drugs such as Axitinib (72), Cetuximab (73), GDC0941 (74), and Gefitinib (52) have been applied to clinical practice. However, the most recent adjuvant clinical trials have not shown any value for adding targeted agents, like cetuximab, to standard chemotherapies in stage III disease, despite improved outcomes in the metastatic setting (75). Additionally, pathologic features (76), MSI (77), Mutations of BRAF, KRAS, and PIK3CA (78), supervised prognostic genomic signatures (79), and unsupervised gene expression molecular subtypes (80) all contribute to the definition of optimal adjuvant treatments for patients. Nevertheless, none of the gene signatures known to date can predict benefits from therapy in COAD (75). In our study, the Wilcoxon rank sum test demonstrated the significant influence of SDHs’ expression level on targeted drug sensitivity, showing the great potential for SDH to predict benefit from therapy in COAD. With the help of SDHs’ expression level, we would predict targeted drug therapy outcomes more precisely. Furthermore, based on the properties of SDHA targeting immune checkpoints to regulate immune infiltration, we believe that SDHA may be a crucial gene in the SDHs family, which plays an essential role in the development of immunotherapy and targeted drug therapy of COAD.

## Conclusions

To sum up, our study comprehensively assessed the expression and prognostic value of SDHs in COAD and explored the pathway mechanisms involved and the immune cell correlations. Our findings suggested that SDHs might be potential biomarkers indicating the prognosis and therapeutic efficacy for patients with COAD and were associated with COAD immune microenvironment.

## Data availability statement

The original contributions presented in the study are included in the article/**Supplementary Materials**, further inquiries can be directed to the corresponding author/s

## Ethics statement

The studies involving human participants were reviewed and approved by Medical Ethics Committee of the Second Affiliated Hospital of Wenzhou Medical University. The patients/participants provided their written informed consent to participate in this study.

## Author contributions

HN and KL designed the study, analyzed the data, and drafted the paper. KL, XX, and WL critically revised it for important intellectual content. HN, JF, WZ, YC, and YG assisted in data acquisition and analysis. RNA extraction, reverse transcription, and qPCR were performed by PG, and WZ. IHC was performed by HN, PG, CH, CZ, and BP. All authors revised the manuscript. HN, PG, and JF contributed equally to this work. All authors contributed to the article and approved the submitted version.
