# Supplementary figures and images for: Comprehensive analysis of the prognosis, tumor microenvironment, and immunotherapy response of SDHs in colon adenocarcinoma
Source: Front Immunol. 2023 Mar 6;14:1093974. doi: 10.3389/fimmu.2023.1093974 (PMC10025334; doi:10.3389/fimmu.2023.1093974)

**Supplementary Figure 1 | (A-F)** The volcano figures of 8 DEGs in CHOL, COAD, LIHC, READ, and STAD.

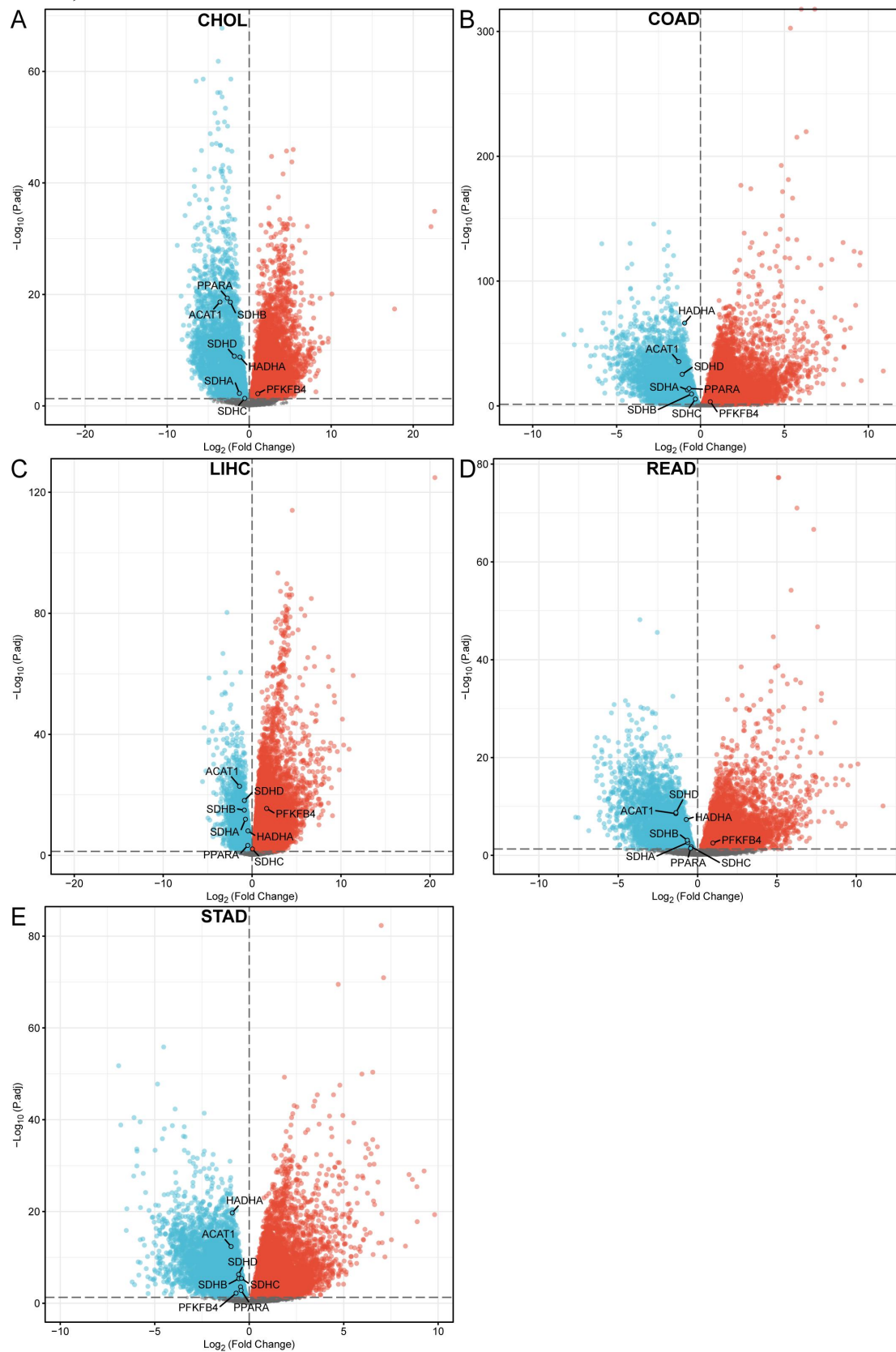

Supplement: Supplementary file 2 [file DataSheet_1.pdf]
