# Supplementary material for: Comprehensive analysis of the prognosis, tumor microenvironment, and immunotherapy response of SDHs in colon adenocarcinoma
Source: Front Immunol. 2023 Mar 6;14:1093974. doi: 10.3389/fimmu.2023.1093974 (PMC10025334; doi:10.3389/fimmu.2023.1093974)

Supplementary Figure 3 | Kaplan-Meier overall survival of ACAT1, HADHA, PFKFB4, and PPARA. **(A-E)** CHOL, COAD, LIHC, READ, and STAD Kaplan-Meier overall survival in indicated tumor types from TCGA database.

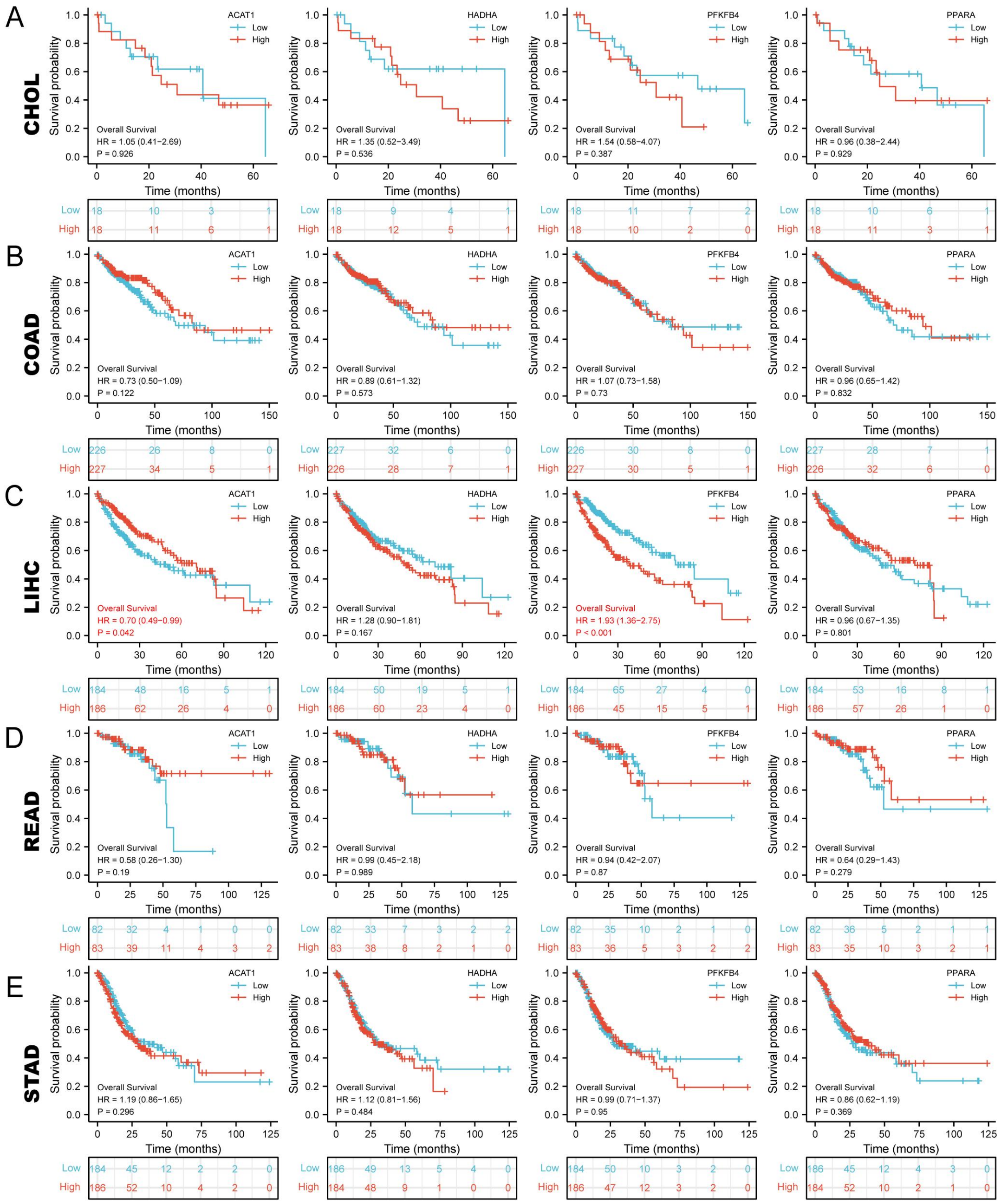

Supplement: Supplementary file 4 [file DataSheet_3.pdf]
