# Supplementary material for: Comprehensive analysis of the prognosis, tumor microenvironment, and immunotherapy response of SDHs in colon adenocarcinoma
Source: Front Immunol. 2023 Mar 6;14:1093974. doi: 10.3389/fimmu.2023.1093974 (PMC10025334; doi:10.3389/fimmu.2023.1093974)

Supplementary Figure 4 | Prognostic significance of SDHs in GSE14333. **(A-D)** Kaplan-Meier overall survival of SDHs in COAD.

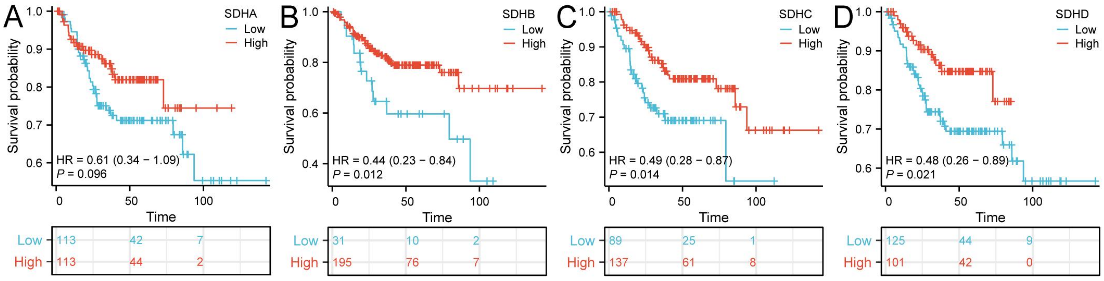

Supplement: Supplementary file 5 [file DataSheet_4.pdf]
