# Supplementary material for: Comprehensive analysis of the prognosis, tumor microenvironment, and immunotherapy response of SDHs in colon adenocarcinoma
Source: Front Immunol. 2023 Mar 6;14:1093974. doi: 10.3389/fimmu.2023.1093974 (PMC10025334; doi:10.3389/fimmu.2023.1093974)

Supplementary Figure 6 | (A) UMAP plots displayed 13 cell-types by tissue types. **(B-E)** UMAP plots demonstrated the expression of SDHs across 13 cell types.

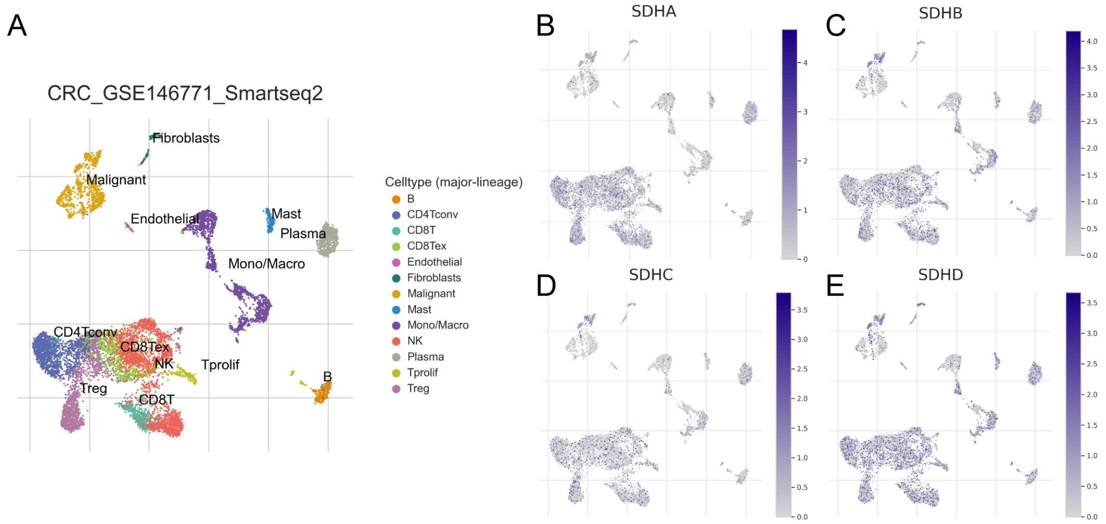

Supplement: Supplementary file 7 [file DataSheet_6.pdf]
