# Supplementary material for: Comprehensive analysis of the prognosis, tumor microenvironment, and immunotherapy response of SDHs in colon adenocarcinoma
Source: Front Immunol. 2023 Mar 6;14:1093974. doi: 10.3389/fimmu.2023.1093974 (PMC10025334; doi:10.3389/fimmu.2023.1093974)
Supplement: Supplementary file 8 [file DataSheet_7.docx]

https://www.jianguoyun.com/p/DS5SxG8Qk9mTCxip0ecEIAA
