# Supplementary material for: Comprehensive analysis of the prognosis, tumor microenvironment, and immunotherapy response of SDHs in colon adenocarcinoma
Source: Front Immunol. 2023 Mar 6;14:1093974. doi: 10.3389/fimmu.2023.1093974 (PMC10025334; doi:10.3389/fimmu.2023.1093974)

Supplementary Figure 5 | (A-D) The ssGSEA analysis based on SDH factor expression for COAD and different types of immune cells. *p < 0.05; **p < 0.01 and ***p < 0.001; ns, not significant.

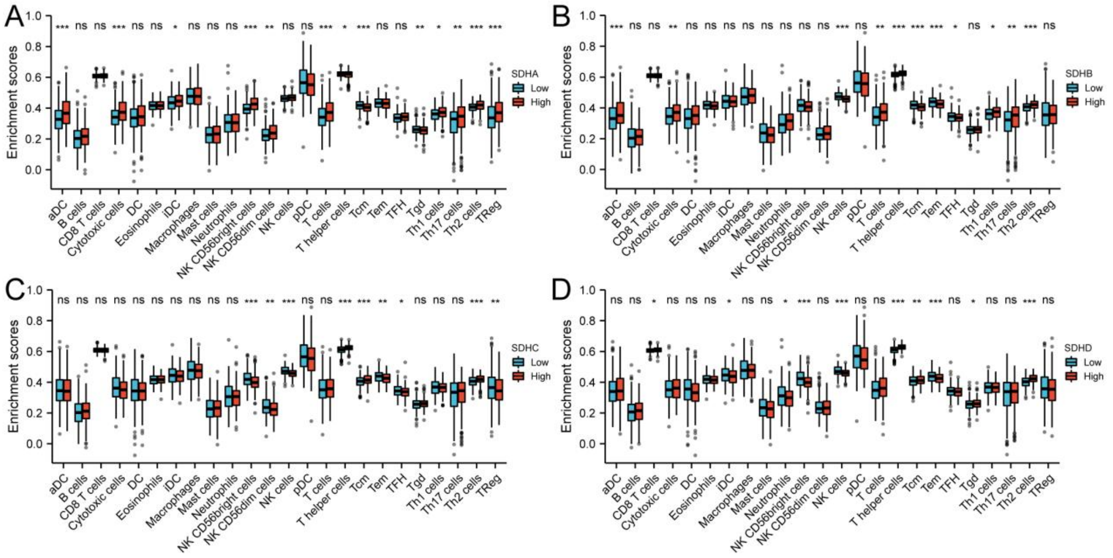

Supplement: Supplementary file 6 [file DataSheet_5.pdf]
